# Application of Ti6Al7Nb Alloy for the Manufacture of Biomechanical Functional Structures (BFS) for Custom-Made Bone Implants

**DOI:** 10.3390/ma11060971

**Published:** 2018-06-08

**Authors:** Patrycja Szymczyk, Grzegorz Ziółkowski, Adam Junka, Edward Chlebus

**Affiliations:** 1 Center for Advanced Manufacturing Technologies (CAMT/FPC), Faculty of Mechanical Engineering, Wroclaw University of Science and Technology, Łukasiewicza 5, 50-371 Wrocław, Poland; grzegorz.ziolkowski@pwr.edu.pl (G.Z.); edward.chlebus@pwr.edu.pl (E.C.); 2 Department of Pharmaceutical Microbiology and Parasitology, Wrocław Medical University, Borowska 211A, 50-556 Wrocław, Poland; feliks.junka@gmail.com (A.J.)

**Keywords:** selective laser melting, Ti6Al7Nb alloy, biomedical functional structures, CT, osteoblasts

## Abstract

Unlike conventional manufacturing techniques, additive manufacturing (AM) can form objects of complex shape and geometry in an almost unrestricted manner. AM’s advantages include higher control of local process parameters and a possibility to use two or more various materials during manufacture. In this work, we applied one of AM technologies, selective laser melting, using Ti6Al7Nb alloy to produce biomedical functional structures (BFS) in the form of bone implants. Five types of BFS structures (A1, A2, A3, B, C) were manufactured for the research. The aim of this study was to investigate such technological aspects as architecture, manufacturing methods, process parameters, surface modification, and to compare them with such functional properties such as accuracy, mechanical, and biological in manufactured implants. Initial in vitro studies were performed using osteoblast cell line hFOB 1.19 (ATCC CRL-11372) (American Type Culture Collection). The results of the presented study confirm high applicative potential of AM to produce bone implants of high accuracy and geometric complexity, displaying desired mechanical properties. The experimental tests, as well as geometrical accuracy analysis, showed that the square shaped (A3) BFS structures were characterized by the lowest deviation range and smallestanisotropy of mechanical properties. Moreover, cell culture experiments performed in this study proved that the designed and obtained implant’s internal porosity (A3) enhances the growth of bone cells (osteoblasts) and can obtain predesigned biomechanical characteristics comparable to those of the bone tissue.

## 1. Introduction

The application of additive manufacturing (AM)—such as SLM (selective laser melting), LENS (laser engineered net shaping), and EBM (electron beam melting)—opens up new possibilities for the design of modern bone and cartilage implants. The novelty of AM technology is owed to its ability to create geometrically advanced forms of implants displaying desired mechanical properties and high biocompatibility with the surrounding living tissues. Great examples are metal and polymer scaffolds of complex structure, containing the patient’s own stem cells seeded inside the implant together with bioactive agents [1,2,3,4,5]. Other examples are implants and prostheses, customized to individual patient anatomy and physiology, often designed on the basis of precise topographic data obtained by means of computed tomography (CT) scans [6].

AM can produce scaffold-like, porous bone implants of high flexibility and decreased weight and density. Another advantage of such biomechanical functional structure (BFS) geometry is an increased (in comparison to traditional implants) transport of tissue fluid within the implant–tissue connections. The manufacture of BFS displaying specific chemical, biological and mechanical properties adjusted to the patient’s actual load, bone deformation and/or displacement is an effective way to provide and restore his mobility and life quality. However, there are presently no coherent data concerning the implant’s optimal structure with regard to tissue overgrowth and differentiation, probably due to considerable differences resulting from the application of various technologies of scaffold manufacturing. Geometric matching of BFS structures with tissues is achieved by the appropriate spatial structures’ porosity and pore sizes—two parameters essential for proper cell migration within the implant. Several studies have shown that pore shape may have a significant impact on cell response and the rate of tissue regeneration. These parameters are believed to increase with the ratio of the implant’s curvature; moreover, cells grow more eagerly on concave surfaces in comparison to convex and planar surfaces. Also, a higher cell growth was observed in implants with a larger number of corners. Tissue formed by osteoblasts is strongly influenced by the geometrical features of channels [7,8,9]. It was shown that minimal BFS porosity providing sufficient cell infiltration and bone ingrowth should be at least 40% [10]. Other researchers estimated this parameter as 55–85% and emphasized the fact that excessive porosity of a BFS may disturb its mechanical properties [11,12].

Another issue to overcome during the design of biocompatibile implants is selecting their optimal pore size. According to some authors [13,14,15,16,17], pore sizes from 50 to 500 μm (and according to some authors even 1200 μm) correlate with a satisfying level of bone regeneration. Importantly, it has been shown that higher porosity results in improved bone regeneration in vivo, while lower porosity is found to be more effective in vitro [7,18]. Additionally, it has been shown that small pore sizes are easily clogged and this clot prevents cells from penetration inside the implant. This phenomenon represents a major technological and manufacturing challenge, especially in the context of metal AM technology [14,19]. In the case of non-resorbable materials, larger pores (<300 µm) enable smooth cell migration [20]. Not only a pore size, but also global architecture has a large impact on cell migration. Straight channels (of square cross-section) and straight connections between individual pores facilitate ingrowth of cells into the implant and allow nutrients to be efficiently propagated within the implant [21].

It is believed that BFS implants should be a surface for new tissue growth and temporarily replace diseased or damaged bone. BFS should bear the loads imposed by daily routine and biological processes. If bone replacement scaffolds with diversified structure are to be used to fill bone loss, they should also display an appropriate level of mechanical strength. It has been established that cortical and cancellous bone have compressive strengths in the ranges of 100–230 MPa and 2–12 MPa, respectively. Their Young’s moduli are in the ranges of 3–30 GPa and 0.02–0.2 GPa, respectively [22]. It is of vital importance to obtain adequately low manufactured structure stiffness in comparison to stiffness of the surrounding bone (it allows to reduce stress shielding at implant–bone contact point) [20]. Moreover, mechanical load on bone tissue at implant–bone contact point is necessary, because it induces bone regeneration, which in turn stimulates bone tissue growth [23]. Selection of material meeting the aforementioned functions is extremely demanding.

Titanium alloys might be the material of choice in this case, however current SLM-based research focuses mainly on Ti6Al4V alloy. Therefore, in this work we focused on an alternative titanium alloy, namely Ti6Al7Nb. It is not commonly used for AM processing. However, it is gaining more and more attention, mainly thanks to its good mechanical parameters, anticorrosive properties, and weak solubility of niobium oxide in the tissue environment resulting in low or no toxicity to bone cells [24,25,26,27]. Moreover, our team has longstanding expertise in analyzing and utilization of Ti6Al7Nb, also as a potential material for implants.

It is presently known that implantation effectiveness depends not only on the implant’s mechanical or functional properties, but also on surface characteristics [28,29]. In the case of such complex spatial structures as BFS, their surface modification cannot be performed by means of traditional techniques (e.g., machining or vibro-abrasive machining), but only by the application of chemical or electrochemical methods. In our previous work on Ti6Al7Nb implants, we observed that the use of the latter method strongly correlated with a decreased ability of *Staphylococcus aureus* and *Pseudomonas aeruginosa* (common bone pathogens) to adhere to cleansed scaffold surfaces. Probably nitrogen and fluorine, elements of known bactericidal ability and used as cleansing agents, were responsible for this phenomenon [30,31].

Bearing in mind the need for developing new and functional implants, the aim of present study was to design, manufacture and investigate the properties of Ti6Al7Nb alloy structures with regard to their functional and biological features and to evaluate their potential application as modern custom-made bone implants. 

## 2. Materials and Methods

### 2.1. Specimen Manufacturing

**Figure 1:**
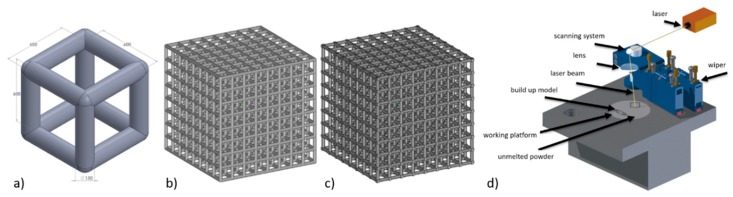
(**a**) Unit cell model of the designed structure of the specimens for technical research, (**b**) designed geometry, (**c**) technological model, (**d**) SLM 50 device.

A computer-aided design (CAD) was created using the Magics software (15.0, Materialise HQ, Leuven, Belgium). A model of a porous unit cell built of struts was designed in the form of a cube (Figure 1a). Strut diameter was set at 150 µm, while the distance between strut axes was 600 µm [26]. The cubic model of the specimen used in the study was composed of 512 unit cells (8 in each direction) that formed a 4.95 × 4.95 × 4.95 mm cube (Figure 1b,c). The porosity of the designed structure was 85%. The designed geometry of BFS specimens is shown in Figure 1.

The specimens were fabricated using ReaLizer SLM 50 (Realizer GmbH, Borchen, Germany) (Figure 1d) from Ti6Al7Nb alloy of 20–63 µm particle diameter (TLS Technik GmbH & Co Spezialpulver KG, Bitterfield, Germany). During the fabrication, an argon inert atmosphere was used and oxygen volume was in the range of 0.2–0.4 ppm. Manufacturing process parameters are shown in Table 1.

**Figure 2:**
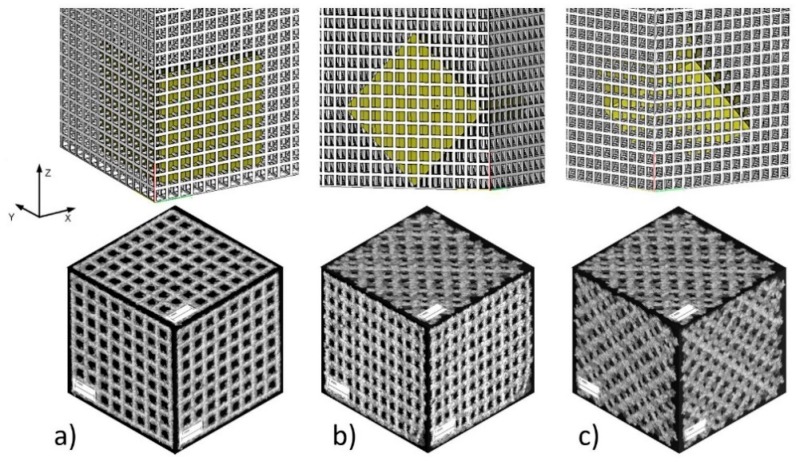
BFS specimen types: (**a**) type A—specimen structure without rotation with perpendicular struts in each plane; (**b**) type B—structure rotated 45° with respect to one axis; (**c**) type C—structure rotated 45° with respect to two axes.

The use of such manufacturing process parameters ensures sufficient material melting and allows to achieve material density close to or equal to 100% [26]. Three variants of BFS specimens featuring diversified internal structures were manufactured (Figure 2). Several proposals were prepared of geometric strut structures that could serve as the scaffold for bone implants. The application of varying orientation of individual struts within the cubic sample aimed to determine the impact of the architecture of the BFS sample relative to the force direction with respect to mechanical properties.

**Figure 3:**
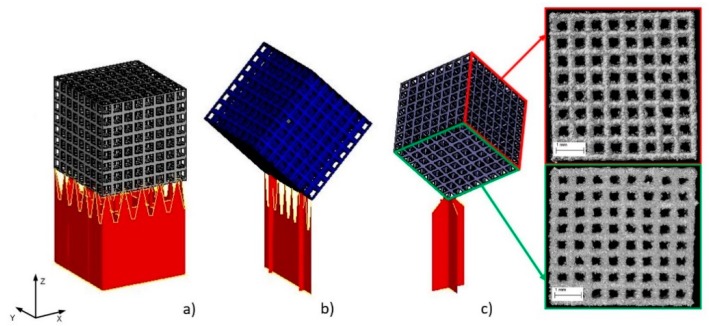
Specimens build orientation: (**a**) type A1—specimen surface parallel to build platform; (**b**) type A2—specimen built on edge; (**c**) type A3—specimen built on corner.

In addition, some specimens (type A) were manufactured in three different orientations with respect to the build direction (Figure 3) to assess the impact of the manufacturing process on the quality of the manufactured structures.

A comprehensive analysis of the samples will determine the relationships between their geometry and mechanical properties, as well as will assess the extent to which the applied process parameters affect the biological and material characteristics of the manufactured structures (BFS).

### 2.2. Evaluation of Mechanical Properties

**Figure 4:**
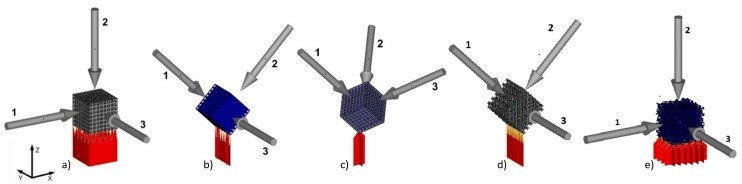
BFS models for analysis and manufactured sample orientation relative to the build platform and marked loading directions for individual samples: (**a**) A1, (**b**) A2, (**c**) A3, (**d**) type B, (**e**) type C.

The manufactured cubic specimens were subjected to static compression test performed with INSTRON 3384 (Instron, Norwood, MA, USA). The tests were carried out at a speed of 0.5 mm/min. Young’s modulus was calculated as the slope of the initial linear range of the stress–strain curve. Compression tests were carried out until the occurrence of the first decrease in stress, which corresponded to the first breakdown of segments (pillars) supporting the subsequent levels of BFS. The maximum substitute stress σ_max_ was calculated by dividing the highest load sustained by the specimen before fracture by the initial cross-sectional area. Samples were analyzed in relation to the direction of force (Figure 4).

### 2.3. Geometric Analysis Using CT

Technical computed tomography was used to record the external and internal geometry of the fabricated structures. The analysis was performed using Zeiss METROTOM 1500 CT system (Carl Zeiss GmbH, Oberkohen, Germany) to enable coordinate measurements of complex geometric structures. All CT measurements were performed at resolution of 21.3 µm. Based on CT reconstruction, an analysis of geometry deviations was performed using GOM Inspect V7.5 (GOM GmbH, Braunschweig, Germany), while wall thickness analysis of the struts was performed using VG Studio Max 2.0 (Volume Graphics, Heidelberg, Germany). Additionally, porosity analysis was performed with Avizo 8.0 (FEI, Hillsboro, OR, USA).

### 2.4. Biological Evaluation

To confirm that BFSs can be effectively used to replace lost bone tissue and particularly to determine the extent of cell adhesion to the surface, initial in vitro studies were performed using osteoblast cell line hFOB 1.19 (ATCC CRL-11372) (American Type Culture Collection, VA, USA). For the purposes of microbiological testing, A3 type samples of cylindrical form have been produced with external dimensions of ø 6.2 × 6 mm. Because of complex internal geometry of the manufactured BFS structures, to improve surface quality and to remove powder grains remaining on the surface of the struts their surface has been chemically etched in accordance with the guidelines presented in our other paper [30]. As in the above-mentioned work, we divided the samples into two groups: non-CP samples—submitted to preliminary cleaning using ultrasonic cleaner containing 99.7% isopropyl alcohol; CP samples—pre-cleaned in 99.7% isopropyl alcohol and then subjected to a chemical polishing in ultrasonic bath with the mixture of 5 mL HF (50%) and 15 mL HNO3 (50%) in 200 mL of ultrapure water.

#### 2.4.1. In Vitro Cytotoxicity of BFS Structures against Osteoblast Cell Line

The extracts were prepared according ISO 10993 standard: biological evaluation of medical devices; Part 5: Tests for in vitro cytotoxicity; Part 12: Biological evaluation of medical devices, sample preparation and reference materials (ISO 10993-5:2009 and ISO/IEC 17025:2005). BFS structures were sterilized at 120 °C. Next, sterile specimens were placed in 1mL of sterile F12 medium (for osteoblasts culture) and left for 24 h/37 °C/5% CO_2_. After incubation, the conditioned medium was used in cytotoxicity evaluation. Briefly, the conditioned media were introduced to osteoblast cell cultures and incubated for 24 h, 48 h, 72 h in 5% CO_2_ at 37 °C. After a specified time of incubation, the medium was removed and 100 µL of NR solution (40 µg/mL; Sigma-Aldrich, St. Louis, MO, USA) was introduced to the wells of the plate. The cells were incubated with NR for 2 h at 37 °C. After incubation, the dye was removed, the wells were rinsed with PBS (Sigma-Aldrich) and left to dry at room temperature. Subsequently, 150 µL of de-stain solution (50% ethanol 96%, 49% deionized water, 1% glacial acetic acid; POCH, Gliwice, Poland) was introduced to each well. The plate was vigorously shaken in a microtiter plate shaker for 30 min until NR was extracted from the cells and formed a homogenous solution. Next, the value of NR absorbance was measured spectrometrically using a microplate reader (Multi-scan GO, Thermo Fisher Scientific, Waltham, MA, USA) at 540 nm wavelength. The absorbance value of cells not treated with extracts was considered 100% of potential cellular growth (positive control).

#### 2.4.2. Osteoblast’s Growth and Multiplication on Implants

1mL containing 1.5 × 105 cfu of osteoblasts was introduced to a well of a 24-well plate containing series I and II BFS structures and incubated for 7, 14, and 30 days at 37 °C/5CO_2_. The medium was changed every 48 h or earlier if it changed color from red to green (internal medium indicator showing pH change). All specimens were cultured in a static culture. The number of adhered cells was counted as presented in Section 2.4.1, and selected samples were dedicated for SEM analysis.

#### 2.4.3. Scanning Electron Microscopy

To assess cell growth on implants, a SEM (Carl Zeiss GmbH, Oberkohen, Germany) analysis was performed. The implants incubated in the presence of osteoblasts for the periods given in Section 2.4.2. Then, they were fixed using 3% glutaraldehyde (POCH) for 15 min at room temperature. Then, the samples were rinsed twice with phosphate buffer (PBS; Sigma-Aldrich, Germany) to remove the fixative. The next step was dehydration in increasing concentrations of ethanol (25%, 60%, 95%, and 100%; POCH) for 5 min in each solution. After rinsing off the ethanol, the samples were dried. Then, the samples were covered with gold and palladium (60:40; sputter current, 40 mA; sputter time, 50 s) using a Q150T Quorum machine (Quorum Technologies Ltd., Laughton, UK) and examined under a Zeiss EVO MA25 scanning electron microscope (Carl Zeiss GmbH, Oberkohen, Germany).

### 2.5. Ability of S. epidermidis to form Biofilm

The impact of surface modifications of Ti6Al7Nb scaffolds on ability of *S. epidermidis* strain was investigated. The *S. epidermidis* strains were cultured on McConkey agar plates and then incubated in the TSB liquid medium (Biocorp, Warsaw, Poland) overnight. Subsequently, the strain liquid culture was diluted to obtain suspension of 2 × 108 cells/mL. The samples were incubated in the bacterial presence for 24 h/37 °C in the aerobic conditions and then vigorously rinsed using 0.9% NaCl and subjected to vortex mixing for 1 min. Bacterial colonies were counted and number of bacterial cells forming biofilm on the implants was assessed. All measures were repeated three times.

### 2.6. Statistical Analysis

Statistical calculations were performed using Statistica software (10, Statsoft, Krakow, Poland) and Mann–Whitney test. Statistical significance was defined as *p* ≤ 0.05.

## 3. Results

### 3.1. Mechanical Properties of BFS

Our studies were conducted in relation to the types of structure (Figure 3) and build orientation as well as loading direction in regard to manufacturing direction (Figure 4). For that purpose 15 specimens from each orientation were analyzed (five for each loading direction). Maximum compression stress of 158 MPa was obtained for type A1 specimens. In this group we also observed the highest anisotropy of values depending on the direction of applied loading force (Table 2), which was respectively: 85 ± 31 MPa (1), 158 ± 14 MPa (2), 82 ± 30 MPa (3).

The most even results were obtained for type A3 specimens for which the determined mean values were 105 ± 4 MPa. Young’s modulus of porous Ti6Al7Nb structure A3 was at the level of 3 GPa, which is between that of trabecular and cortical bone and similar for those achieved in the literature [32]. Such a manner of sample orientation on the build platform enables the achievement of uniform geometric deviations and the highest relative values of equivalent compressive strength. The results have shown that the mechanical properties of the manufactured structures are not accidental and depend on load direction and manufacturing method. Porous BFSs feature sufficient mechanical strength and low stiffness. Changing the dimensions of elementary cells (pores), strut thickness, and their orientation enables to scale the structures in relation to the designed equivalent strength of the structure and to optimize it in terms of clinical requirements.

### 3.2. CT Reconstruction

**Figure 5:**
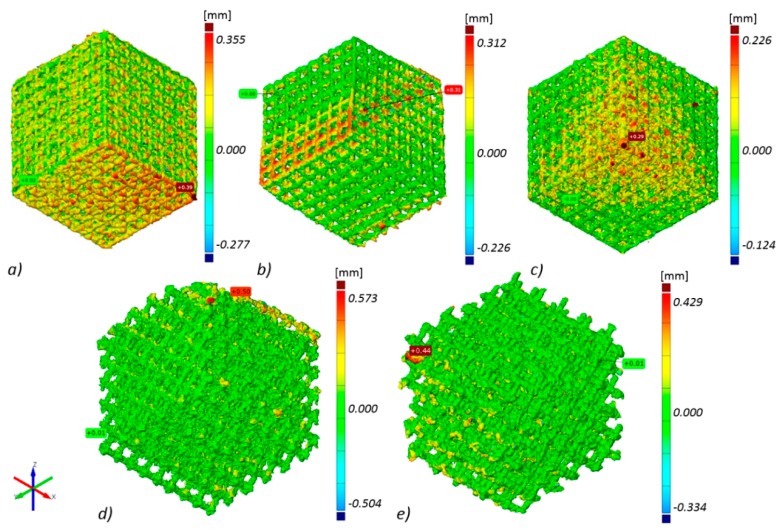
Geometry deviations of actual models from nominal model: (**a**) ‘A1 type’ specimen, (**b**) ‘A2 type’ specimen, (**c**) ‘A3 type’ specimen, (**d**) ‘B type’ specimen, (**e**) ‘C type’ specimen (the most appropriate scale was used for each sample so the geometric deviations were easy to analyze and compare).

Thanks to technical computed tomography (CT) reconstruction, the internal geometry of the fabricated BFSs was evaluated. A-type samples were subjected to testing as they displayed the best mechanical performance and show geometry-dependent anisotropy of properties. During the first stage of the CT analysis, the geometry of the designed BFS structures was compared with the actual geometry of the fabricated structures. The actual models (reconstructed, STL format) were fitted to the nominal models (designed, CAD format) using the ‘best-fit’ method. The above enabled to determine the deviations between design geometry and models obtained on the basis of CT reconstruction for the manufactured elements in differing positions relative to the build platform (Figure 5).

**Figure 6:**
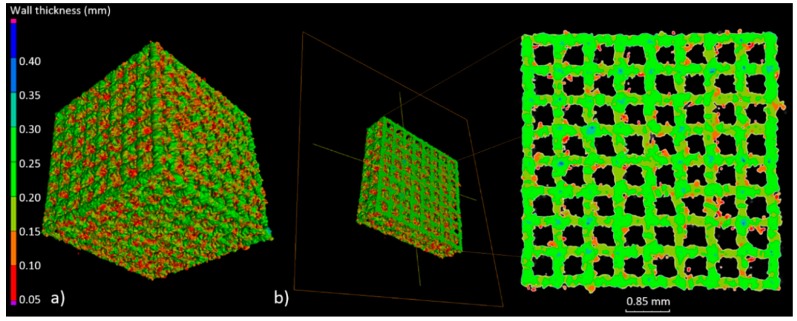
(**a**) Example of wall thickness analysis (‘A3 type’ specimen); (**b**) cross-section showing the thickness changes of the struts in the A3 specimen.

The highest geometry deviations (in the range of −504 to 573 µm and −334 to 429 µm) were observed for B and C type specimens respectively. For ‘A1 type’ specimens majority of the deviations were revealed within limits −227 to 355 µm. The lowest deviations range were displayed for A2 (−226 to 312 µm) and for A3 (−124 to 226 µm) type (Figure 5). Based on the results obtained at this stage, it was decided that for further testing, A-type samples—characterized by the highest manufacturing accuracy—would be used. Thickness analysis of the fabricated BFS struts showed that the largest diameters were noted for struts oriented horizontally on the build platform. It was also revealed that they were nearly twice as thick as the design values. The wall thickness of the struts in ‘A3 type’ specimen with the lowest geometry deviations was in the range between 100 and 250 µm (Figure 6). The mean diameter of the struts in this case was at the level of 225 µm.

**Figure 7:**
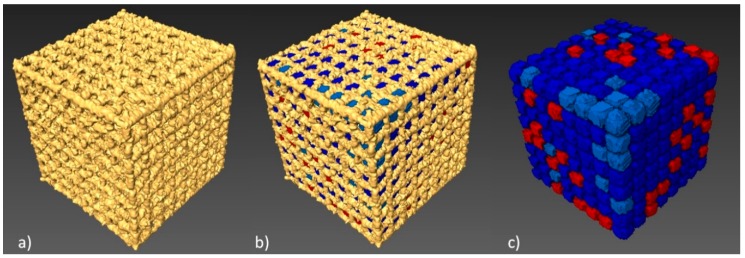
Porosity analysis of BFS structure: (**a**) CT image thresholding for BFS structure geometry, (**b**) BFS structure with porosity values, (**c**) pores after the separation process with colors indicating their differing volumes.

Next, we determined total porosity and volume of individual pores of the specimen with the lowest geometry deviations. The geometry of the examined BFS type A3 structure and the volume of the individual pores was determined (Figure 7).

**Figure 8:**
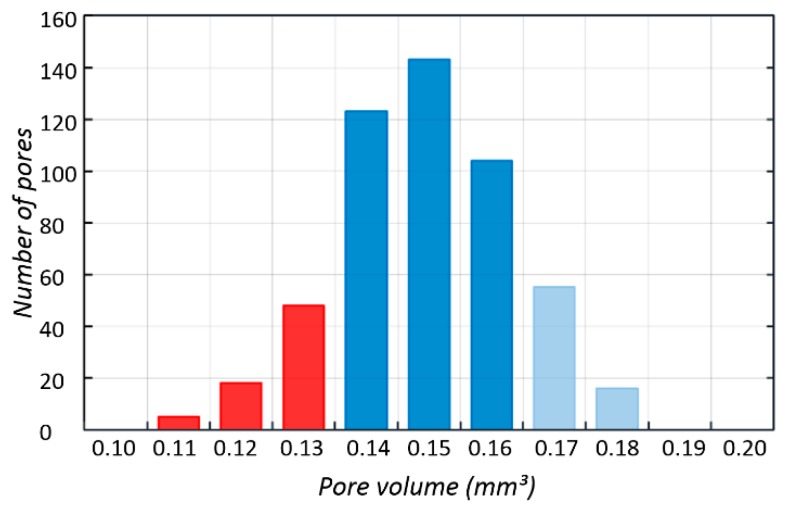
Histogram of pore volume distribution obtained using Avizo 8.0.

512 pores were registered; the smallest of which had a volume of 0.11 mm³, while for the largest—0.18 mm³—the nominal volume of a single pore was 0.2 mm³. Our analysis has shown that all identified pores had a smaller actual volume. However, it should be noted that no blocked pores were identified and, consequently it has been confirmed that all channels are fully unobstructed. Figure 8 shows a histogram of pore volume distribution.

On the basis of the information obtained about the volume of individual pores, porosity of the fabricated structure was determined, on the basis of Equation (1).

(1) $$P=1-\frac{V_{BFS}}{V_{BFS}+V_{P}\;}$$

where $V_{BFS}$ is the volume of the BFS structure and $V_{P}$ is the pore volume. Table 3 includes a summary of the analysis of BFS structure geometry.

Total porosity of the fabricated structure was 56% and it was 29% lower than the porosity planned in the design. This discrepancy was related to the geometry of the fabricated struts whose diameters, due to the nature of the SLM process, were higher than those designed. The achieved porosity suggests that bone cells may be more prone to grow into the pores, which will be investigated further below.

### 3.3. In-Vitro Cell Response

**Figure 9:**
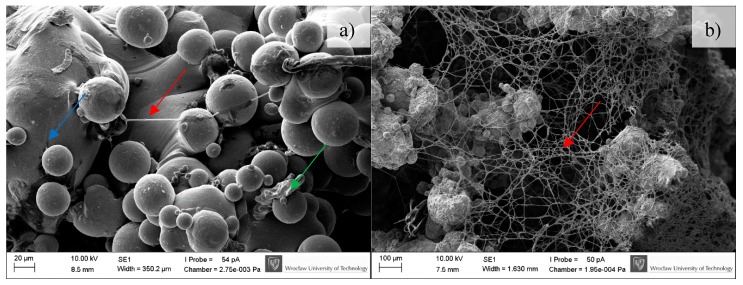
Osteoblasts after seven days of incubation on the tested implants. Samples not subjected (non-CP samples) (**a**) and subjected to chemical polishing (CP samples) (**b**). Blue arrow indicates BFS material; green arrow indicate adhered cells, while red ones extracellular matrix produced by osteoblasts, SEM.

**Figure 10:**
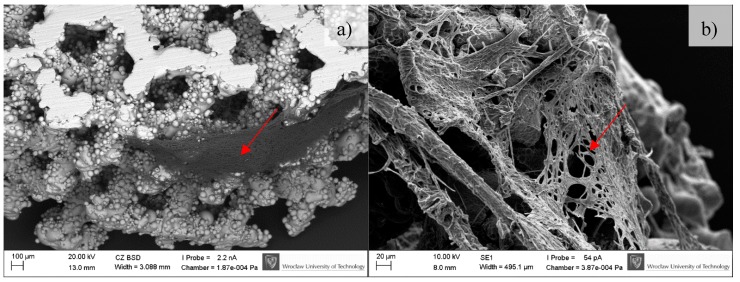
Osteoblasts after 14 days of incubation on tested implants. Samples non-subjected (non-CP samples) (**a**) and subjected to chemical polishing (CP samples) (**b**). Red arrows indicate increasing (please compare it to Figure 9) amount of extracellular matrix produced by osteoblasts, SEM.

**Figure 11:**
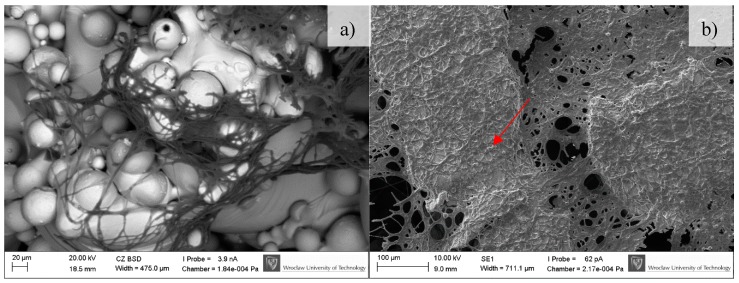
Osteoblasts after 30 days of incubation on tested implants. Samples not subjected (non-CP samples) (**a**) and subjected to chemical polishing (CP samples) (**b**). Red arrow indicate increasing amount of extracellular matrix embedding osteoblasts, SEM.

BFS cytotoxicity was evaluated using NRU cytotoxicity assay procedure as per PN-EN ISO 10993-5, for specimens incubated under controlled conditions (37 °C/5% CO_2_) at 100% humidity under static conditions. The lack of toxicity of the investigated implantation materials was confirmed also in macroscopic observation of the behavior of cells cultured in the presence of TiAl7Nb alloy on which a clear increase in culture density after 48 h and progressing colonization of the area of the investigated sample in comparison with the image obtained after 24 h of incubation. Biological studies revealed that BFSs manufactured using SLM technology have low toxicity and high survival of osteoblasts equal to 75.4% and 90.5%, before (non-CP samples) and after chemical polishing (CP samples), respectively. To evaluate the growth and development of cells on the tested implants, a SEM analysis was conducted. Osteoblasts were visualized on all tested surfaces (Figure 9, Figure 10 and Figure 11). The number of cells changed with time. The longer the cell culture lasted, the more cells were seen on the analyzed fields of observation.

**Figure 12:**
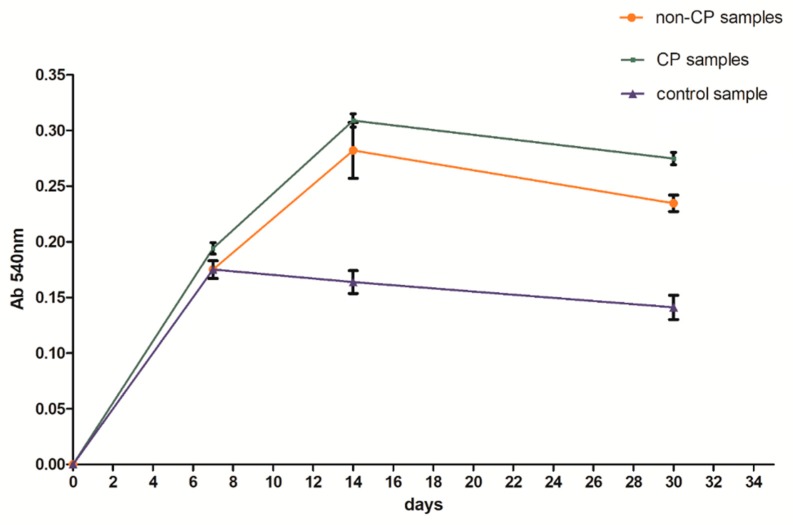
Number of cells with regard to analyzed series of specimens.

A clear difference between the analyzed series may be observed. Samples subjected to chemical polishing (b) were settled by osteoblasts more eagerly than those not subjected to chemical polishing. This trend was observed for every analyzed time point of incubation (7, 14, and 30 days). Since the BFS samples subjected to chemical polishing displayed a higher value of design porosity (total porosity of the structure was 61%, so the chemical etching process applied caused a 5% increase in structure porosity relative to the initial value), the osteoblasts had more surface to adhere to and propagate within. On the other hand, there was a high amount of loosely adhered powder in the samples not subjected to polishing, which probably prevented the adherence of osteoblasts. CFU-osteoblasts, calculated by the NR test, increased systematically with the time of incubation (Figure 12).

Regardless of the time point analyzed, the number of cells was higher for the samples tested in comparison to the control sample. After 14 days, the number of cells on BFS structures exceeded the number of cells in the control sample by 70–90%, while after 30 days of incubation this value was 75–100% higher in comparison to the control sample. The obtained result suggests that the BFS accelerates the osteoblastic differentiation and can promote the formation of extracellular matrix at later stage.

### 3.4. Microbiological Tests

**Figure 13:**
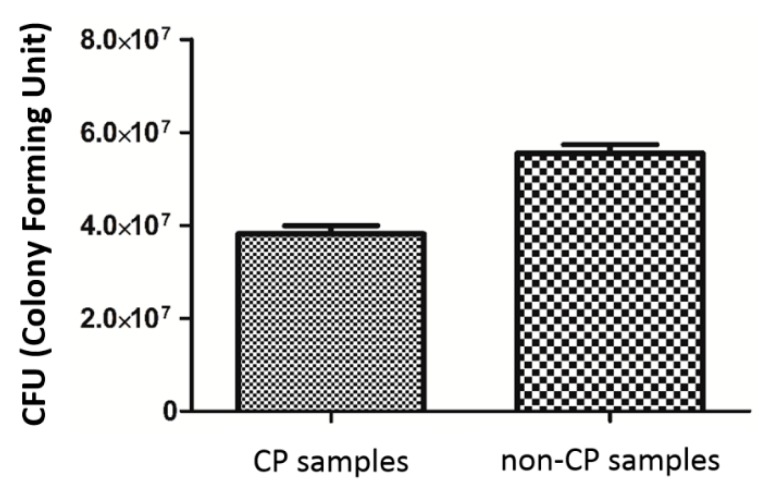
*S. epidermidis* biofilm formation on non-CP and CP samples.

The analyzed strain was able to form strong biofilm structures on both types of scaffold, regardless of the condition of the surface. However, quantitative cultures revealed a significantly decreased number of microbial cells on surface of BFS subjected to chemical polishing (CP samples) (Figure 13). (KW test, *p* < 0.05).

### 3.5. Example of Application

**Figure 14:**
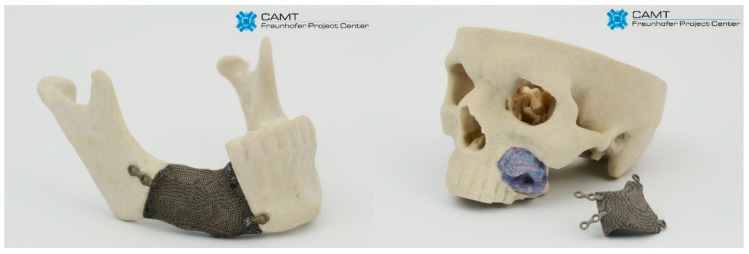
Examples of custom-made implants made of Ti6Al7Nb alloy filled with BFS.

The capabilities, based on processing biocompatible metal alloys, to produce objects with diversified structure, designed and manufactured to support the growth of functional bone tissue with geometries defined by computer 3D models, have created a potential for solving many problems in implantology. Based on CT data, the degree of bone tissue damage and the size and shape of the bone resection was determined. Then, obtained external geometry of the bone defect was filled with the replicated BSF structure. According to obtained results, we have chosen the ‘A3 type’ sample as they display the best geometrical accuracy and do not show geometry-dependent anisotropy of properties. Examples of customized jaw and facial implants for oncological treatment designed on the basis of the results of this study are shown in Figure 14.

## 4. Discussion

Ensuring an appropriate interaction between the implant and its biological environment poses a huge challenge. That is why the properties of biomaterials—such as non-toxicity, corrosion resistance or controlled degradability, and mechanical and biological characteristics—have for a long time been regarded as crucial in terms of selection of an appropriate biomaterial to a specific biomedical application. Permanent and irreversible damage to bone tissue has motivated research on filling the cavities with biomaterials. The above fillings are intended to replace the pulp and serve as a suitable foothold for new tissue growth. The above structures should have a porous architecture, enabling the penetration of the implant by bone-forming osteoblasts, which in time should enable the provision of blood supply and innervation of the filled cavity.

The AM (additive manufacturing) process enables the manufacture of homogenous functional structures (scaffolds) having certain chemical, biological, and mechanical properties suited to the expected actual load, deformation, and displacement resulting from an individual’s anatomy and physiology. Artificial bone scaffolds can be used in tissue engineering and can be helpful in the treatment of large bone cavities. Our results indicate the suitability of SLM technology for the manufacture of biomechanical functional structures from Ti6Al7Nb alloy that can precisely fill the bone loss. The mechanical properties obtained in our study are similar to those obtained for specimens of similar geometry and porosity reported in the literature [33,34]. Depending on structure orientation, its dimensions, architecture, and SLM process parameters, our experimentally determined substitute modulus of elasticity was within the range from 485 to 3038 MPa, at the value of substitute compressive strength from 28 to 158 MPa. On the basis of the above, we can claim that it is possible to predict and suitably design material characteristics—such as compressive strength, stiffness, or maximum load a structure can carry—by changing its geometric features. In line with the results reported in the literature, the highest strength of materials manufactured using SLM, not subjected to heat treatment, is observed for the build direction [24]. The above can also be observed in the case of BFSs analyzed in this paper for which the highest compressive stress was observed for type A1 specimens when the force was applied in the build direction. It was revealed that shape deviations which cause a disproportion of thickness of individual struts result in varying mechanical properties obtained for different directions of load application. Geometrical verification of the fabricated structures was made using the CT method which enabled us to analyze and describe the manufactured structures in a non-invasive manner [35]. A digital model facilitates the determination of strut thickness, porosity of the global structure and material, comparison of the actual and nominal geometry, determination of unit cell geometry parameters (diameter, the size of contact points of unit cells), and determination of the surface area-volume ratio. The results are very precise and provide information on the quality of the manufactured biomechanical functional structures and confirm the high accuracy of the manufacturing process.

The observed shape deviations on the reconstruction images occur mainly in the bottom layers, which is directly associated with the specific features of additive manufacturing. Thus, due to the largest surfaces which are parallel to the build platform, the highest deviations were measured for the specimen built parallel to the build platform (type A1 specimen). In the case of type A3 specimens, due to the structure being inclined to the built platform evenly at all sides and the struts parallel to the built platform being eliminated, the deviations are similar independently of their direction, and the manufactured struts have similar diameters.

Material surface plays an extremely important role in the response of the biological environment to an artificial medical device. For BFSs fabricated using the AM process, surface modification through chemical etching enables to reduce the number of powder particles attached to the surface and consequently to improve their surface quality. Additionally, such untreated surfaces are not always suitable for biomedical applications and often specific surface properties need to be ensured to achieve biological integration and bone formation. Chemical polishing described in this work and used for surface modification of BFS scaffolds caused an increased amount of nitrogen and fluorine on the scaffolds surface [30,31]. Nitrided layers are characterized by high resistance to abrasive wear, which significantly improves tribological properties of the fabricated structures [36]. Additionally, it creates a barrier, reducing the penetration of hydrogen into the material structure. Fluorine on the other hand (in limited doses) stimulates bone metabolism and restructuring [37]. This type of surface also displays antibacterial properties and significantly prevents the growth of *S. epidermidis* strain on the implant surface, which was also confirmed by previous results of the authors’ research on staphylococcal and pseudomonas cells.

Biological studies revealed that BFSs manufactured using SLM technology have low toxicity, which is consistent with the literature data [38]. CFU-osteoblast colonies, calculated using the NR test, increased systematically with time of incubation and after 30 days of incubation it was 75–100% higher than the control sample. This is also confirmed by literature research which shows that the discs made of Ti6Al7Nb manufactured with the use of SLM technology are biologically well tolerated, without producing any adverse reactions [39]. The condition and size of the culture as well as the morphology of human osteoblasts during contact with the surface of a BFS sample indicate that the conditions for implant incorporation into the recipient tissues listed in the introduction can be met.

The results suggest that the designed and manufactured BFSs are ideal for replacing bone tissue loss. By enabling cell growth due to their open porosity, they effectively integrate with the surrounding bone tissue. Moreover, the analyzed BFSs were manufactured from Ti6Al7Nb titanium alloy which is characterized by low cytotoxicity. The next step of this investigation should undoubtedly be analysis of osteoblast gene expression and protein level to estimate true nature of interaction between cell and implant of specific porosity/geometry. However, it should be noted that the base-line results on cytotoxicity and colonization ratio presented in this manuscript are of a highly promising nature.

## 5. Conclusions

The application of additive technologies opens up new possibilities for the design of modern implants in the form of metallic scaffolds (BFS). Both their geometry and programmed mechanical characteristics rely directly on the data obtained from medical CT scans. Based on the analysis of five types of BSF structures (A1, A2, A3, B, C) made of titanium alloy, we observed the correlation between applied technological parameters and obtained functional features. The mechanical tests as well as geometrical accuracy analysis showed that the square shaped (A3) BFS structures were characterized by the lowest deviations range and smallest anisotropy of mechanical properties. Additionally, CT examinations revealed that the (A3) structure is characterized by open porosity with simple connections between particular pores however, in vitro tests indicate the best method of post-processing treatment (chemical polishing) enabling better cell adhesion capacity.

The results of the present study confirm the potential of the selective laser melting method for producing custom-made implants from Ti6Al7Nb titanium alloy featuring not only high accuracy and high geometric complexity but also controllable mechanical properties such as strength or stiffness. The achieved distribution of internal porosity facilitates the growth of osteoblasts and enables the achievement of predesigned biomechanical characteristics matching those of the bone tissue.

## Figures and Tables

**Table 1:** Process parameters used to manufacture test specimens

Process Parameter	Laser Power	Spot Size	Scan Velocity	Layer Thickness	Overlap	Scanning Strategy
unit	W	µm	mm/s	µm	%	-
value	25	100	200	50	30	XY

**Table 2:** Process parameters used to manufacture test specimens

Force Direction	Parameters	BFS Types				
		A1	A2	A3	B	C
**1**	σ [MPa]	85 ± 31	50 ± 6	100 ± 3	32 ± 5	29 ± 5
	E [MPa]	2430 ± 727	1752 ± 671	2860 ± 174	855 ± 130	817 ± 108
**2**	σ [MPa]	158 ± 14	51 ± 6	107 ± 4	29 ± 2	29 ± 9
	E [MPa]	743 ± 270	2139 ± 173	3009 ± 202	817 ± 195	648 ± 78
**3**	σ [MPa]	82 ± 30	28 ± 7	108 ± 5	81 ± 2	28 ± 8
	E [MPa]	2193 ± 867	485 ± 112	3303 ± 145	2356 ± 129	790 ± 102

**Table 3:** Results of BFS structure geometry analysis using CT

BFS Type A3	Strut Diameter µm	Total Porosity P %	Volume of Single Pore mm^3^	Total Surface mm^2^
CAD model	150	85%	0.202	431.34
CT data	225	56%	0.150	849.49

## References

[1] Ryan G., Pandit A., Apatsidis D.B. (2006). Fabrication methods of porous metals for use in orthopaedic applications. Biomaterials.

[2] Otsuki B., Takemoto M., Fujibayashi S., Neo M., Kokubo T., Nakamura T. (2006). Pore throat size and connectivity determine bone and tissue ingrowth into porous implants: Three-dimensional micro-CT based structural analyses of porous bioactive titanium implants. Biomaterials.

[3] Kumaresan T., Gandhinathan R., Ramu M., Ananthasubramanian M., Banu Pradheepa K. (2016). Design, analysis and fabrication of polyamide/hydroxyapatite porous structured scaffold using selective laser sintering method for bio-medical applications. J. Mech. Sci. Technol..

[4] Temple J.P., Hutton D.L., Hung B.P., Huri P.Y., Cook C.A., Kondragunta R., Jia X., Grayson W.L. (2014). Engineering anatomically shaped vascularized bone grafts with hASCs and 3D-printed PCL scaffolds. J. Biomed. Mater. Res. A.

[5] Melchels F.P.W., Domingos M.A.N., Kleina T.J., Maldaa J., Bartoloc P.J., Hutmacher D.W. (2012). Additive manufacturing of tissues and organs. Prog. Polym. Sci..

[6] Stuyts B., Peersman G., Thienpont E., Van den Eeden E., Van der Bracht H. (2015). Custom-made lateral femoral hemiarthroplasty for traumatic bone loss: A case report. Knee.

[7] Zadpoor A.A. (2015). Bone tissue regeneration: The role of scaffold geometry. Biomater. Sci..

[8] Bidan C.M., Kommareddy K.P., Rumpler M., Kollmannsberger P., Fratzl P., Dunlop J.W. (2013). Geometry as a factor for tissue growth: Towards shape optimization of tissue engineering scaffolds. Adv. Healthc. Mater..

[9] Rumpler M., Woesz A., Dunlop J.W., van Dongen J.T., Fratzl P. (2008). The effect of geometry on three-dimensional tissue growth. J. R. Soc. Interface.

[10] Bragdon C.R., Jasty M., Greene M., Rubash H.E., Harris W.H. (2004). Biologic fixation of total hip implants. Insights gained from a series of canine studies. J. Bone Jt. Surg..

[11] Tuchinskiy L., Loutfy R. (2003). Titanium foams for medical applications. Medical Device Materials, Proceedings of the Materials &Processes for Medical Devices Conferences, Anaheim, CA, USA, 8–10 September 2003.

[12] Levine B.R., Sporer S., Poggie R.A., Della Valle C.J., Jacobs J.J. (2006). Experimental and clinical performance of porous tantalum in orthopaedic surgery. Biomaterials.

[13] Manjubala I., Woesz A., Pilz C., Rumpler M., Fratzl-Zelman N., Roschger P., Stampfl J., Fratzl P. (2005). Biomimetic mineral-organic composite scaffolds with controlled internal architecture. J. Mater. Sci. Mater. Med..

[14] Warnke P.H., Douglas T., Wollny P., Sherry E., Steiner M., Galonska S., Becker S.T., Springer I.N., Wiltfang J., Sivananthan S. (2009). Rapid prototyping: Porous titanium alloy scaffolds produced by selective laser melting for bone tissue engineering. Tissue Eng. Part C Methods.

[15] Harrysson O.L.A., Cansizoglu O., Marcellin-Little D.J., Cormier D.R., West H.A. (2008). Direct metal fabrication of titanium implants with tailored materials and mechanical properties using electron beam melting technology. Mater. Sci. Eng. C.

[16] Hollander D.A., von Walter M., Wirtz T., Sellei R., Schmidt-Rohlfing B., Paar O., Erlia H. (2006). Structural, mechanical and in vitro characterization of individually structured Ti-6Al-4V produced by direct laser forming. Biomaterials.

[17] Lopez-Heredia M.A., Goyenvalle E., Aguado E., Leroux C., Dorget M., Layrolle P. (2006). Bone Growth in Porous Titanium Implants made by Rapid Prototyping. Key Eng. Mater..

[18] Karageorgiou V., Kaplan D. (2005). Porosity of 3D biomaterial scaffolds and osteogenesis. Biomaterials.

[19] Xue W., Krishna B.V., Bandyopadhyay A., Bose S. (2007). Processing and biocompatibility evaluation of laser processed porous titanium. Acta Biomater..

[20] Tan X.P., Tan Y.J., Chow C.S.L., Tor S.B., Yeong W.Y. (2017). Metallic powder-bed based 3D printing of cellular scaffolds for orthopaedic implants: A state-of-the-art review on manufacturing, topological design, mechanical properties and biocompatibility. Mater. Sci. Eng. C.

[21] Goodman S., Toksvig-Larsen S., Aspenberg P. (1993). Ingrowth of bone into pores in titanium chambers implanted in rabbits: Effect of pore cross-sectional shape in the presence of dynamic shear. J. Biomed. Mater. Res..

[22] Wang X., Xu S., Zhou S., Xu W., Leary M., Choong P., Qian M., Brandt M., Xie Y.M. (2016). Topological design and additive manufacturing of porous metals for bone scaffolds and orthopaedic implants: A review. Biomaterials.

[23] Van Cleynenbreugel T., Van Oosterwyck H., Vander Sloten J., Schrooten J. (2002). Trabecular bone scaffolding using a biomimetic approach. J. Mater. Sci. Mater. Med..

[24] Chlebus E., Kuźnicka B., Kurzynowski T., Dybała B. (2011). Microstructure and mechanical behaviour of Ti-6Al-7Nb Alloy produced by selective laser melting. Mater. Charact..

[25] Leordean D., Marcu T., Radu S.A., Berce P. (2011). Porous metal structures from Ti alloys produced by SLM technology. Acad. J. Manuf. Eng..

[26] Pawlak A., Szymczyk P., Ziółkowski G., Dybała B., Chlebus E. (2015). Fabrication of microscaffolds from Ti-6Al-7Nb alloy by SLM. Rapid Prototyp. J..

[27] Brunette D.M., Tengvall P., Textor M., Thomsen P. (2001). Titanium in Medicine.

[28] Kerckhofs G., Van Bael S., Pyka G., Schrooten J., Moesen M., Loeck D., Wevers M. Non-destructive characterization of the influence of surface modification on the morphology and mechanical behavior of rapid prototyped Ti6Al4V bone tissue engineering scaffolds. Proceedings of the European Conference for Non-Destructive Testing.

[29] Sitting C., Textor M., Spencer N.D., Wieland M., Vallotton P.H. (1999). Surface characterization of implant materials c.p. Ti, Ti-6Al-7Nb and Ti-6Al-4V with different pretreatments. J. Mater. Sci. Mater. Med..

[30] Szymczyk P., Junka A., Ziółkowski G., Bartoszewicz M., Smutnicka D., Chlebus E. (2013). The ability of S.aureus to form biofilm on the Ti-6Al-7Nb bone scaffolds produced by Selective Laser Melting and subjected to the different types of surface modifications. Acta Bioeng. Biomech..

[31] Junka A.F., Szymczyk P., Secewicz A., Pawlak A., Smutnicka D., Ziółkowski G., Bartoszewicz M., Chlebus E. (2016). The chemical digestion of Ti6Al7Nb scaffolds produced by Selective Laser Melting reduces significantly ability of Pseudomonas aeruginosa to form biofilm. Acta Bioeng. Biomech..

[32] Miao G., Xiang L. (2016). Development of porous Ti6Al4V/chitosan sponge composite scaffold for orthopedic applications. Mater. Sci. Eng. C.

[33] Parthasarathy J., Starly B., Raman S., Christensen A. (2010). Mechanical evaluation of porous titanium (Ti6Al4V) structures with electron beam melting (EBM). J. Mech. Behav. Biomed. Mater..

[34] Lin C.-Y., Wirtz T., LaMarca F., Hollister S.J. (2007). Structural and mechanical evaluation of topology optimized titanium interbody fusion cage fabricated by selective laser melting process. J. Biomed. Mater. Res..

[35] Ziółkowski G., Szymczyk P., Dybała B., Chlebus E., Pawlak A. (2015). Geometric Characteristics of Scaffolds Made by Additive Manufacturing. Powder Metall. Met. Ceram..

[36] Liua X., Chub P.K., Ding C. (2004). Surface modification of titanium, titanium alloys, and related materials for biomedical applications. Mater. Sci. Eng..

[37] Ellingsen J.E. (1995). Pre-treatment of titanium implants with fluoride improves their retention in bone. J. Mater. Sci. Mater. Med..

[38] Rumiński S., Noga M., Ostrowska B., Pawlak A., Dybała B., Dąbrowski B., Święszkowski W., Lewandowska-Szumieł M. (2013). Osteogenic-like behaviour of adipose derived stem cells in selected scaffolds obtained by 3D-printing. Eur. Cells Mater..

[39] Rotaru H., Armencea G., Spîrchez D., Berce C., Marcu T., Leordean D., Seong-Gon K., Sang-Woon L., Dinu C., Băciu G. (2013). In vivo behavior of surface modified Ti alloys used in selective laser melting for custom-made implants. A preliminary study. Rom. J. Morphol. Embryol..

